# Improved hypertension control at six months using an adapted WHO HEARTS-based implementation strategy at a large urban HIV clinic in Uganda

**DOI:** 10.1186/s12913-022-08045-8

**Published:** 2022-05-25

**Authors:** Martin Muddu, Fred C. Semitala, Isaac Kimera, Mary Mbuliro, Rebecca Ssennyonjo, Simon P. Kigozi, Rodgers Katwesigye, Florence Ayebare, Christabellah Namugenyi, Frank Mugabe, Gerald Mutungi, Chris T. Longenecker, Anne R. Katahoire, Isaac Ssinabulya, Jeremy I. Schwartz

**Affiliations:** 1 Makerere University Joint AIDS Program (MJAP), P.O. Box 7072, Kampala, Uganda; 2 Makerere University College of Health Sciences, Kampala, Uganda; 3 Infectious Diseases Research Collaboration (IDRC), Kampala, Uganda; 4 Ministry of Health, Kampala, Uganda; 5 Case Western Reserve University School of Medicine, Cleveland, OH USA; 6 Uganda Heart Institute, Mulago Hospital Complex, Kampala, Uganda; 7 Yale School of Medicine, 333 Cedar Street, New Haven, CT 06511 USA

**Keywords:** Integrated care for hypertension and HIV, Using an adapted WHO HEARTS strategy

## Abstract

**Objectives:**

To adapt a World Health Organization HEARTS-based implementation strategy for hypertension (HTN) control at a large urban HIV clinic in Uganda and determine six-month HTN and HIV outcomes among a cohort of adult persons living with HIV (PLHIV).

**Methods:**

Our implementation strategy included six elements: health education, medication adherence, and lifestyle counseling; routine HTN screening; task shifting of HTN treatment; evidence-based HTN treatment protocol; consistent supply of HTN medicines free to patients; and inclusion of HTN-specific monitoring and evaluation tools. We conducted a pre-post study from October 2019 to March 2020 to determine the effect of this strategy on HTN and HIV outcomes at baseline and six months. Our cohort comprised adult PLHIV diagnosed with HTN who made at least one clinic visit within two months prior to study onset.

**Findings:**

We enrolled 1,015 hypertensive PLHIV. The mean age was 50.1 ± 9.5 years and 62.6% were female. HTN outcomes improved between baseline and six months: mean systolic BP (154.3 ± 20.0 to 132.3 ± 13.8 mmHg, *p* < 0.001); mean diastolic BP (97.7 ± 13.1 to 85.3 ± 9.5 mmHg, *p* < 0.001) and proportion of patients with controlled HTN (9.3% to 74.1%, *p* < 0.001). The HTN care cascade also improved: treatment initiation (13.4% to 100%), retention in care (16.2% to 98.5%), monitoring (16.2% to 98.5%), and BP control among those initiated on HTN treatment (2.2% to 75.2%). HIV cascade steps remained high (> 95% at baseline and six months) and viral suppression was unchanged (98.7% to 99.2%, *p* = 0.712). Taking ART for more than two years and HIV viral suppression were independent predictors of HTN control at six months.

**Conclusions:**

A HEARTS-based implementation strategy at a large, urban HIV center facilitates integration of HTN and HIV care and improves HTN outcomes while sustaining HIV control. Further implementation research is needed to study HTN/HIV integration in varied clinical settings among diverse populations.

## Introduction

### Background

Across low- and middle-income countries (LMIC), integrating the screening and treatment of hypertension (HTN) into existing HIV clinical care is widely recommended [1–5], yet its implementation remains suboptimal [6–8]. Integrated HTN/HIV care involves provision of both services for HTN and HIV in synchronized visits to the same clinic. This approach is more patient-centered than disease-specific or “vertical” programs and minimizes duplication and fragmentation of services, thus increasing efficiency [9]. Since HTN contributes the greatest population attributable risk for cardiovascular disease (CVD) among persons living with HIV (PLHIV) [10, 11], it is of great priority in efforts to integrate non-communicable diseases (NCDs) care with that of chronic HIV [4, 9]. There is an urgent need therefore to leverage the HIV infrastructure to provide contextually appropriate integrated HTN/HIV services in LMICs for dual control [12].

The World Health Organization (WHO) HEARTS technical packages for HTN management provide guidelines for HTN care on: healthy lifestyle, evidence-based protocols, access to HTN medicines, CVD risk assessment, task shifting and monitoring and evaluation [13–17]. Six-month control of blood pressure among people treated for HTN is a HEARTS Core Indicator [18]. The HEARTS tools are useful in the implementation of high-quality public health HTN management in LMIC settings. Though promising in improving HTN control among the general population, the effectiveness of WHO HEARTS in facilitating the integration of HTN care in the HIV clinical setting has not been studied [19, 20].

We recently conducted a mixed methods study at a large, urban HIV clinic (hereafter referred to as “Mulago ISS”) in Uganda which had achieved all the three UNAIDS 90–90-90 goals of initiating ART for PLHIV, retention in care, and viral suppression [21, 22]. In this clinic, there is a high prevalence of HTN (24.3%) among adult PLHIV. However, only one percent of these patients had been initiated on HTN treatment, 15% of those were retained in care, and, subsequently HTN control was achieved among only five percent [21]. We identified the barriers and facilitators of integrating HTN management into HIV care using qualitative methods, guided by the Capability, Opportunity, Motivation and Behavior (COM-B) model [21, 23, 24], which specifies that changing any behavior requires changing capability, opportunity and/or motivation to perform the desired behavior [23, 24]. Despite great interest in integrated HTN/HIV care from patients and providers as well as optimal patient adherence to available antiretroviral treatment (ART), the following were key barriers: suboptimal provider knowledge and skills for HTN care, lack of HTN treatment protocols, limited access to HTN medications and inadequate systems for monitoring and evaluation of HTN care [21].

Building upon these identified barriers, we undertook a systematic process to develop a HEARTS-based implementation strategy for HTN/HIV integration targeting both healthcare providers and patients [13]. This process involved using the Behavior Change Wheel (BCW) which is an intervention development framework that provides a comprehensive model of behavior change. It uses the COM-B model to understand behavior [13–17]. We mapped the barriers of HTN/HIV integration identified in our previous study onto the COM-B domains. The BCW framework was used to identify intervention functions to address barriers within each COM-B domain and to identify evidence-based behavior change techniques to carry out the intervention functions [23, 24]. We then adapted components of the HEARTS technical packages for cardiovascular control. Herein, we describe the baseline and six-month clinical outcomes of HTN and HIV among a prospective cohort of patients with both conditions who were provided integrated care at Uganda’s largest HIV clinic.

## Methods

### Study design

This was a pre-post study conducted between October 2019 and March 2020 to determine HTN and HIV treatment outcomes at baseline and six months after introducing a HEARTS-based implementation strategy for HTN/HIV integration at Mulago ISS.

### Study setting

We conducted the study at Mulago ISS, a large HIV clinic in Kampala, Uganda that provides comprehensive HIV services to over 16,500 PLHIV. The clinic is located within the Mulago National Referral and Teaching Hospital Complex and is owned and operated by the Makerere University Joint AIDS Program (MJAP). HIV clinical services at Mulago ISS include HIV testing and counseling and provision of ART. All available HIV medicines and services at the clinic are provided at no cost to the patients.

In line with the Uganda National Guidelines for HIV care [25, 26], PLHIV at Mulago ISS are routinely screened for HTN. Prior to study commencement, the clinic had already achieved universal screening for HTN among all PLHIV during each clinic visit and had surpassed the 95% target of initiating ART among PLHIV. However, the clinic had no stock of HTN medications. Identified hypertensive PLHIV were given prescriptions to buy HTN medications from commercial pharmacies away from the HIV clinic. MJAP uses an electric medical records (EMR) system to facilitate clinical data capture and documentation.

This study was funded by Resolve to Save Lives, through its Learning, Implementation, Networking, Knowledge and Support (LINKS) program.

### Developing the HEARTS-based implementation strategy for integrated HTN/HIV care

The implementation strategy, guided by the BCW, was developed through a series of four stakeholder meetings. Sixteen participants were purposively selected for these meetings, including two nurses, one pharmacist, one pharmacy technician, one laboratory technician, one clinic administrator, two social scientists and two monitoring and evaluation officers from the study site. Other participants included two HIV physicians, two cardiologists, and two Ministry of Health (MoH) NCD policy experts.

In the first two meetings, two investigators with expertise in implementation science (MM and RK) oriented meeting participants to the BCW framework, the nominal group technique for brainstorming [27], and the Affordability, Practicality, Effectiveness, Acceptability, Side effects, Equity (APEASE) criteria [28], for prioritizing implementation strategies.

Through brainstoming, meeting partcipants first identified intervention functions to address barriers to integrating HTN and HIV care. The following intervention functions were identified: education, training, enablement, persuasion, modeling, and environmental restructuring (Table 1). Using a scoring system of 1–6, the meeting participants applied the APEASE criteria to each intervention function to assess its appropriateness to integrated HTN/HIV treatment in Uganda [29]. After identifying the intervention functions, the team used the behavior change technique (BCT) taxonomy of the BCW to identify BCTs that best matched the selected intervention functions. BCTs are the active ingredients of an implementation strategy (Table 1) [30]. The team then brainstormed on the modes of delivery for the multi-component implementation strategy by adapting HEARTS components (Table 1). To determine local relevance, feasibility, and acceptability of the proposed implementation strategy**,** the last two stakeholder meetings reviewed the proposed modes of delivery to further refine the strategy. They achieved consensus on the relative importance and feasibility of each strategy thus contextualizing them to Ugandan HIV clinics. The multicomponent implementation strategy for integrated HTN/HIV care is described below and summarized in Table 1.

**Table 1:** Targeted barriers and components of the implementation strategy to integrate HTN care into Mulago ISS clinic: Adapted from WHO HEARTS technical packages for cardiovascular disease control

Barriers targeted	COM-B Domain	Intervention function	Behavior change technique (BCT)	Mode of delivery
**Component 1: Health education, medication adherence, and lifestyle counseling**				
Patient lack of knowledge of HTN risk, complications and self-management	Psychological capability	Education	Information about health consequences of HTN	Health education by HTN/HIV peer educators
Healthcare providers and patients lack knowledge of HTN-HIV drug interactions	Psychological capability	Education	Information about health consequences of HTN	Health education by nurse and dispenser
Patients prioritize adherence to ART over HTN medications	Reflective motivation	Persuasion	Information about health consequences of HTN	Adherence counseling for both anti-hypertensives and ART by nurse
**Component 2: Routine HTN screening during each clinic visit**				
Inadequate supply of automated BP machines	Physical opportunity	Environmental restructuring	Adding objects to the environment	Provide 2 automated BP machines to HIV clinic
**Component 3: Task shifting of HTN screening and treatment**				
HTN/HIV peer educators’ skills to measure BP	Physical capability	Training	Demonstration of the behavior	Nurses to mentor HTN/HIV peer educators to measure BP
HTN prescriptions are mainly done by doctors; limited task shifting to clinical officers and nurses	Social opportunity	Modeling	Demonstration of the behavior	Train nurses on HTN treatment through CME and mentoring
**Component 4: Evidence based HTN treatment protocol**				
Lack of simple evidence-based treatment protocol for HTN	Physical opportunity	Enablement	Social support	Provide stepwise evidence-based treatment protocol to all clinical rooms
**Component 5: Consistent supply of HTN medicines free to patients**				
Lack of on-site HTN medications despite demand from patients and providers	Physical opportunity	Enablement	Social support	Pharmacy assistant to make timely orders of protocol anti-hypertensive medicines
Cost of buying anti-hypertensive medicines is high; patients can’t afford	Physical opportunity	Enablement	Social support	Provide anti-hypertensive medicines to patients at no cost
**Component 6: Inclusion of HTN-specific monitoring and evaluation tools**				
Lack of monitoring indicators for HTN	Psychological capability	Enablement	Goal setting	Apply WHO HTN monitoring indicators and mentor providers on them
Lack of performance targets and review of HTN care quality	Automatic motivation	Persuasion	Feedback on behavior	Develop and share quarterly targets with providers. Monthly performance review meetings
Lack of data collection tools and data bases for HTN care	Physical opportunity	Enablement	Social support	Integrate HTN data elements into HIV data collection tools and EMR. Print and avail HTN register and patient treatment cards

*CME* Continuing medical education, *EMR* Electronic medical records

The strategy includes six major components:

1. **Health education, medication adherence, and lifestyle counseling**
  The HTN/HIV care protocol provided guidance to healthcare providers on health and lifestyle education for HTN. Health education to patients was provided by nurses and PLHIV peer educators and focused on possible side effects of medicines and their management as well as the need for and implementation of physical exercise, healthy diet, salt reduction, weight reduction, and smoking cessation. Nurses and dispensers provided on the job mentoring to providers and health education to patients on identification and management of side effects for hypertension medications as well as drug- drug interactions. In addition, the protocol emphasized adherence counseling and support for both HTN and HIV medicines.
2. **Routine HTN screening during each clinic visit**
  Clinic nurses trained PLHIV peer educators on BP measurement, which was performed using validated, automated Omron M6 BP machines [31]. Clinicians repeated the BP measurement for patients whose initial BP was > 140/90 mmHg. Though the clinic had already achieved optimal HTN screening, this was included in the implementation strategy to ensure continued attention to it by all providers.
3. **Task shifting of hypertension treatment**
  In addition to the aforementioned task shifting of screening practices, one doctor trained clinical officers and nurses on HTN medicine prescribing using the protocol in two-hour training sessions. These were followed by ongoing, one-on-one mentoring twice monthly to ensure protocol adherence and safe prescribing practices. We printed and provided a copy of the protocol to each clinician. In addition, two technical experts of the Department of NCDs at MoH provided quarterly support supervision on adherence to the protocol.
4. **Evidence-based HTN treatment protocol**
  We adapted HEARTS recommendations to develop our protocol which provided a simple, stepwise approach to the titration of amlodipine, valsartan, and hydrochlorothiazide as the first-, second-, and third-line therapies, respectively (Fig. 1) [13–17]. In addition to this stepwise approach, the protocol also included guidance for patients in higher risk groups.
5. **Consistent supply of HTN medicines free to patients**
  The choice of medicines was based on availability and local evidence of efficacy [32]. For the purpose of this study, we procured amlodipine, valsartan, and hydrochlorothiazide from a private not for-profit pharmaceutical access program [33, 34], endorsed and regulated by the Uganda MoH. The procurement cost to the study for these medicines ranged from 0.30USD to 1.50USD per patient per month and we provided them to patients at no cost. Outside the pharmaceutical access program, the cost of our protocol medicines in Uganda would have been approximately 10 times higher.

**Fig. 1:**
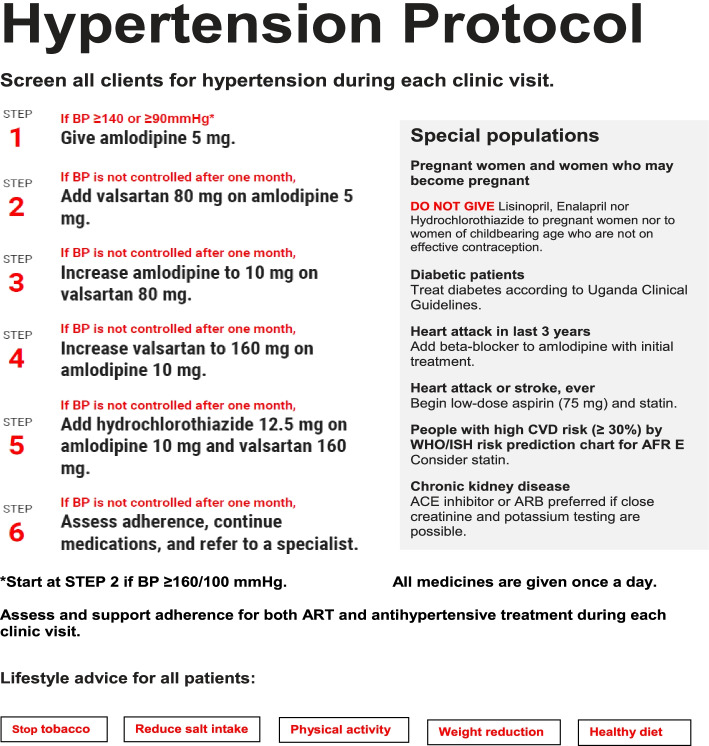
The hypertension management protocol at MJAP
6. **Inclusion of HTN-specific monitoring and evaluation tools**
  In our prior work, we had defined HTN monitoring indicators that parallel those used extensively in HIV care: Screening, Diagnosis, Initiation of treatment, Retention, Monitoring and Control [35]. Monitoring indicators are useful in evaluating the quality of health services and identifying gaps that should be targeted for continuous quality improvement [36]. In addition, we developed and shared quarterly targets on HTN care indicators with healthcare providers during bimonthly and quarterly performance review meetings. We adapted the HEARTS HTN register and CVD patient cards and distributed them to clinic providers. The CVD card was used to record the patent’s anthropometric measurements, comorbidities including diabetes, kidney disease, prior stroke and heart attack; blood pressure and blood sugar values; HTN medicines prescribed; ART regimen; laboratory findings including serums electrolytes, creatinine, urine protein, total cholesterol; and examination of the fundus and the foot. Each patient’s data was entered into both the CVD card and HTN register during each clinic visit.

### Sampling of the study cohort

**Fig. 2:**
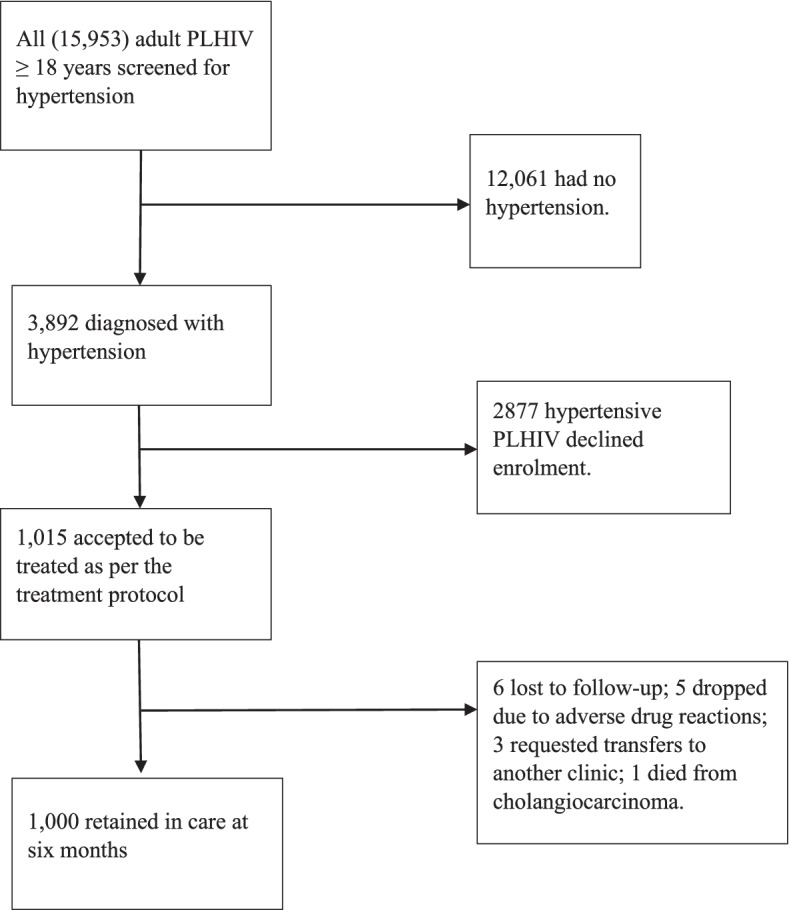
Flow chart of patient enrolment into integrated HTN/HIV Care

By August 1^st^, 2019, the clinic had 15,953 adult PLHIV aged ≥ 18 years. All these were screened for HTN and 3,892 (24.4%) were diagnosed with HTN. For the prospective cohort, we included all hypertensive PLHIV aged 18 years and above enrolled in HIV care at Mulago ISS who made at least one clinic visit between August 1^st^ and September 30^th^, 2019. Study participants provided informed consent to be treated according to the adapted HEARTS protocol. Of the 3,892 hypertensive PLHIV, 1,015 patients accepted to be treated as per the treatment protocol and were enrolled into the study. The other 2,877 hypertensive PLHIV declined enrollment. For the 1,015 patients enrolled into integrated HTN/HIV treatment, we determined both HTN and HIV outcomes at baseline and followed them up for six months after introducing the adapted HEARTS implementation strategy. Of the 15 patients who were not retained at six months, six were lost to follow-up; five opted out due to side effects of HTN medications; three requested transfers to other HIV clinics and one died. At the end of six months, 1000 patients were retained in integrated HTN/HIV care (Fig. 2). We then compared HTN and HIV outcomes at baseline and six months after introducing the implementation strategy.

### Data collection

We prospectively extracted data from the EMR. The EMR at Mulago ISS clinic was adopted from the Uganda MoH open medical records system, which managed patient data on HIV care [21]. The EMR was adapted to include blood pressure and anthropometric values and HTN medicines prescribed. We recorded data on indicators at every stage of the HTN and HIV care cascades. We developed the data collection tools to obtain information on the HTN and HIV care cascade at the Mulago ISS clinic and specifically mapped out the expected baseline and six-month outcomes at each cascade step according to national HIV guidelines and WHO recommendations [25, 26]. Throughout the study, we defined hypertension as “having a documented blood pressure (BP) ≥ 140/90 mmHg or documented use of HTN medications or documented history of hypertension” [35]. We described each cascade step as a proportion of the preceding step and reported them as follows for HTN and HIV: screened, diagnosed, treated, retained, monitored and controlled (Fig. 2). For all proportions, the denominator was the absolute number for each preceding cascade step. The primary outcome was HTN control (BP < 140/90 mmHg at the most recent clinic visit).

### Data analysis

We described baseline characteristics using means and standard deviations for continuous variables, and frequencies and percentages for categorical variables. We compared baseline characteristics of patients who were enrolled and those that were not enrolled into integrated HTN/HIV care.

We then compared the HTN and HIV outcomes at baseline and six months after introducing the implementation strategy in a cohort of hypertensive PLHIV who received integrated HTN/HIV care using a paired t-test statistic and paired sample proportions tests. We determined the six-month trend of the percentage of patients with controlled HTN from baseline to six months after introducing the implementation strategy. To determine proportions along the HTN and HIV care cascades, we conducted descriptive analyses and obtained frequencies and percentages of patients at each defined step compared with the preceding step.

We used a multivariate random effects logistic regression model to determine which baseline characteristics predicted HTN control at six months. We adjusted for age, sex, baseline BP, ART duration, baseline CD4 count, BMI, and baseline ART regimens. We analyzed the data using Stata (version 13). We followed the STROBE guideline in developing this manuscript [35].

## Results

### Baseline characteristics of study participants

Between August and September 2019, we enrolled a cohort of 1,015 hypertensive PLHIV into integrated HIV-hypertension with a mean age of 50.1 ± 9.5 years. Of these, 62.6% were female. Mean baseline systolic and diastolic BP were 154.3 ± 20.0 mmHg and 97.7 ± 13.1 mmHg respectively. A total of 270 (26.6%) participants were obese (body mass index > 30). Overall, the mean CD4 cell count before starting ART was 325.7 ± 251.7 cells per mm^3^. Both cohort participants and non-enrolled hypertensive patients had similar gender compositions. Cohort participants were older, had higher baseline BP, lower baseline CD4 counts, had been on ART longer and had higher BMIs compared to non-enrolled patients (Table 2).

**Table 2:** Characteristics of hypertensive PLHIV at baseline (*N* = 3892)

Characteristics	Patients not enrolled in integrated HTN/HIV care (*N* = 2877)	Patients enrolled in integrated HTN/HIV care (*N* = 1015)	*P* value
Sex			
Male	1,136 (39.5%)	380 (37.4%)	0.250
Female	1,741 (60.5%)	635 (62.6%)	
Mean Age (years)	44.7 ± 10.1	50.1 ± 9.5	< 0.001
Age groups (years)			
18–29	154 (5.4%)	6 (1.0%)	< 0.001
30–39	801 (27.8%)	123 (12.1%)	
40–49	1,038 (36.1%)	361 (35.6%)	
50 and older	884 (30.7%)	525 (51.7%)	
Mean baseline BP (mmHg)			
Systolic ± SD	143.0 ± 15.1	154.3 ± 20.0	< 0.001
Diastolic ± SD	92.6 ± 9.3	97.7 ± 13.1	< 0.001
ART duration			
< 2 years	588 (20.5%)	47 (4.6%)	< 0.001
2—5yrs	775 (27.0%)	215 (21.2%)	
5—10yrs	1,224 (42.6%)	481 (47.4%)	
> 10yrs	288 (10.0%)	272 (26.8%)	
Mean baseline CD4 count	367.5 ± 296.8	325.7 ± 251.7	< 0.001
Baseline CD4 count categories			
< 50	366 (12.7%)	113 (11.1%)	< 0.001
50—< 100	216 (7.5%)	64 (6.3%)	
100—< 200	378 (13.1%)	192 (18.9%)	
> 200	1,917 (66.6%)	646 (63.6%)	
BMI(Kg/m.^2^)			
Underweight (< 19.0)	261 (9.6%)	55 (5.4%)	< 0.001
Normal weight (19.0—< 25.0)	1,237 (45.3)	353 (34.8%)	
Overweight (25.0—< 30.0)	729 (26.7%)	337 (33.2%)	
Obese (> = 30.0)	503 (18.4%)	270 (26.6%)	
Baseline ART regimen			
AZT-3TC-NVP	974 (33.9%)	431 (42.5%)	< 0.001
AZT-3TC-EFV	347 (12.1%)	188 (18.5%)	
TDF-3TC-NVP	224 (7.8%)	96 (9.5%)	
TDF-3TC-EFV	1,010 (35.1%)	240 (23.6%)	
Other	320 (11.1%)	60 (5.9%)	

*HTN* Hypertension, *BP* Blood pressure, *ART* Antiretroviral therapy, *BMI* Body mass index

### Comparison of hypertension and HIV outcomes in the cohort at baseline (before introducing the HEARTS implementation strategy) and at six months of follow up

In this cohort, the mean systolic BP improved from 154.3 ± 20.0 mmHg at baseline to 132.3 ± 13.7 mmHg at six months (*p* < 0.001). Likewise, the mean diastolic BP improved from 97.7 ± 13.1 mmHg at baseline to 85.3 ± 9.5 mmHg at six months (*p* < 0.001). There was a significant improvement in HTN control from 9.3% at baseline to 74.1% at six months, (*p* < 0.001). HIV viral suppression remained high, 98.7% at baseline and 99.2% at six months (*p* = 0.712) (Table 3).

**Table 3:** Comparison of hypertension and HIV outcomes at baseline and six months among patients enrolled in integrated HTN/HIV care

Outcome	Baseline	6 months (*N* = 1015)	*P*-value
Mean systolic BP, mmHg (± SD)	154.3 ± 20.0	132.4 ± 13.8	< 0.001
Mean diastolic BP, mmHg (± SD)	97.7 ± 13.1	85.3 ± 9.5	< 0.001
Patients with controlled hypertension, N (%)	94 (9.3)	752 (74.1)	< 0.001
Patients with controlled HIV (%)	1002 (98.7)	1007 (99.2)	0.712

**Fig. 3:**
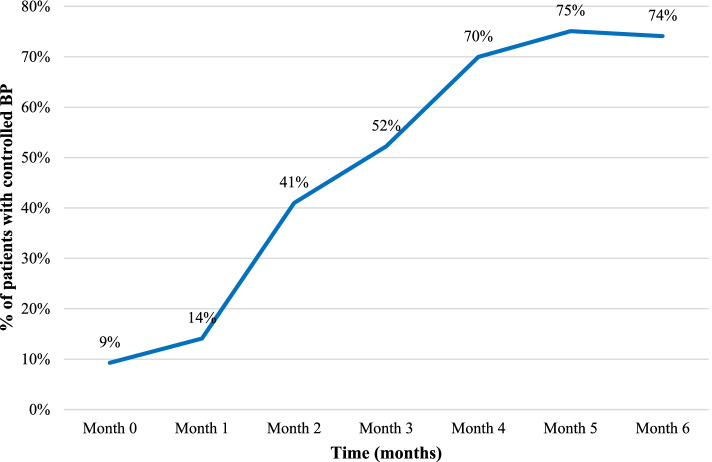
Percentage of patients enrolled in integrated HTN/HIV care with controlled BP (*N* = 1015)

Additionally, there was a positive trend of hypertension control for six months after introducing the adapted HEART implementation strategy (Fig. 3).

The denominator at each time point was 1015 patients who were enrolled into the cohort.

### Baseline HIV Care

**Fig. 4:**
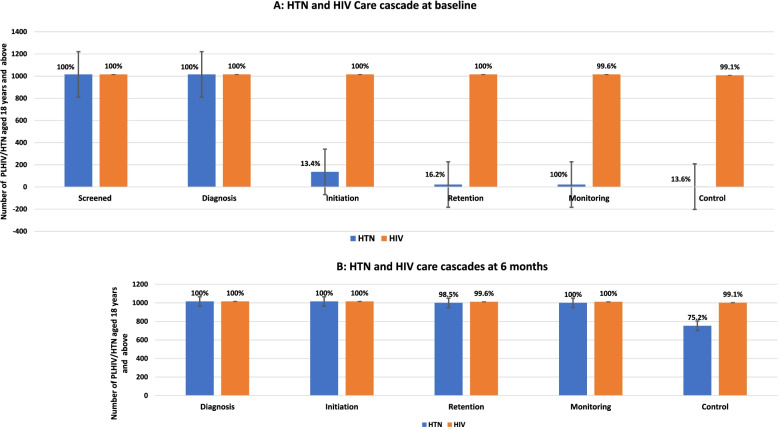
HTN and HIV care cascades at baseline (**A**) and six months (**B**)

At baseline, all the 1,015 cohort participants had been tested for HIV, diagnosed and initiated on ART. All the 1,015 (100%) participants had been retained in care in the last six months and had viral load monitoring of whom 1,007 (99.2%) achieved HIV control (viral suppression) (Fig. 4A).

The HTN and HIV care cascades in Fig. 4 above are for 1,015 hypertensive PLHIV who were enrolled in the cohort. The cascade at baseline was assessed through a retrospective study. Throughout the cascades, the denominator was the absolute number at each preceding step. Definitions of cascade steps: Screening = Number and % of patients who received an HIV test or whose blood pressure was measured before diagnosis; Diagnosis = number and % of patients who were diagnosed with HIV or HTN; Initiation = number and % of patients who were started on ART or HTN medications; Retention = number and % of patients who consistently received ART or HTN medications for six months: Monitoring = number and % of patients who had a viral load test or blood pressure measured while on HTN treatment and Control = number and % of patients with HIV viral suppression (viral load < 1000 copies/ml) or BP < 140/90 mmHg. The error bars indicate the 95% confidence intervals.

### Baseline hypertension care

All the 1,015 cohort participants had been screened and diagnosed for HTN. Of these, 136 (13.4%) were initiated on HTN treatment and given only one agent, amlodipine. Of those initiated on HTN treatment, 22(16.2%) were retained and monitored for HTN. Only three (2.2% of those initiated on treatment) patients achieved HTN control (Fig. 4A). Expressed as a proportion of the total cohort, only 0.3% of patients were controlled.

### HIV care cascade among PLHIV with HTN enrolled into the cohort at six months

All 1,015 PLHIV with HTN who were enrolled into integrated HTN/HIV care had been initiated on ART and HTN medicines, of whom 1,011 (99.6%) were retained in HIV care at six months and had a timely viral load test, with 1,002 (99.1%) achieving HIV control (viral suppression) (Fig. 4B). Expressed as a proportion of the total cohort, 98.7% of enrolled patients had HIV viral suppression at six months.

### HTN care cascade among PLHIV with HTN at six months

All 1,015 (100%) cohort participants were initiated on HTN treatment and 1,000 (98.5%) were retained in HTN care at six months. All those retained were monitored for HTN at six months and 752 (75.2%) achieved HTN control (Fig. 4B). Expressed as a proportion of total cohort, 74.1% of enrolled patients achieved a blood pressure of < 140/90 at six months. Of the patients with controlled HTN at six months, 151 (20%) were on one protocol medicine (amlodipine 5 mg), 587 (78%) were on more than one protocol medicine, and 12 (2%) were on non-protocol hypertension medicines (Table 4).

**Table 4:** Antihypertensive medicine use at baseline and 6-months

Categories	Baseline (*N* = 1015)	Six-months	
		**Controlled (*N* = 752)**	**Not Controlled (*N* = 248)**
**# of antihypertensive medicines**			
0	976 (96.2%)	-	-
1	39 (3.8%)	163 (21%)	12 (5%)
> = 2	-	587 (79%)	236 (95%)
**Protocol medicines and daily dose**			
Amlodipine 5 mg	28	533 (90%)	61 (10%)
Amlodipine 10 mg	11	216 (54%)	182 (46%)
Valsartan 80 mg	-	569 (79%)	149 (21%)
Valsartan 160 mg	-	21 (19%)	88 (81%)
Hydrochlorothiazide 12.5 mg	-	-	-
**Medicine combination by Protocol step**			
**Step 1**. Amlodipine 5 mg	-	151 (20%)	6 (2%)
**Step 2**. Amlodipine 5 mg + Valsartan 80 mg	-	381 (50%)	55 (22%)
**Step 3.** Amlodipine 10 mg + Valsartan 80 mg	-	186 (25%)	93 (38%)
**Step 4**. Amlodipine 10 mg + Valsartan 160 mg	-	20 (3%)	88 (35%)
** Step 5**. Amlodipine 10 mg + Valsataran 160 mg + Hydrochlorothiazide 12.5 mg	-	-	-
Other	-	12 (2%)	6 (2%)

Data for 15 patients was not complete at six months

### Independent predictors of HTN control at six months of treatment

Out of numerous physical, demographic, and clinical characteristics examined, the only independent predictors of HTN control at six months were being on ART for longer than two years. These individuals were two to four times more likely than those with shorter ART durations to achieve HTN control at six months. The likelihood of hypertension control increased with ART duration. Additionally, individuals who had a suppressed HIV viral load were 3.2 times more likely to achieve HTN control compared to those with a non-suppressed viral load (Table 5).

**Table 5:** Predictors of HTN control at six months among participants enrolled in integrated HTN/HIV care (*N* = 1015)

Characteristics	Unadjusted Odds ratios	[95% CI]	Adjusted Odds ratios	[95% CI]
Age groups (years)				
18–29	1		1	
30–39	0.360	0.054 – 2.368	0.280	0.042 – 1.83
40–49	0.311	0.048—1.994	0.219	0.034 – 1.402
50 and older	0.393	0.061 – 2.512	0.275	0.043 – 1.757
Sex				
Female	1		1	
Male	0.961	0.731 – 1.263	0.919	0.687 – 1.229
ART duration				
< 2 years	1		1	
2—5yrs	2.346	1.185 – 4.646	2.294^a^	1.118 – 4.707
5—10yrs	2.612	1.365 – 4.999	2.848^a^	1.377 – 5.890
> 10yrs	3.590	1.835 – 7.025	4.352^a^	2.003 – 9.457
Baseline CD4 count categories				
< 50	1		1	
50—< 100	1.306	0.676 – 2.524	1.190	0.616 – 2.297
100—< 200	1.303	0.791 – 2.145	1.269	0.773 – 2.084
> 200	1.166	0.759 – 1.791	1.172	0.763 – 1.799
HIV viral load				
> = 1000	1		1	
< 1000	3.532	1.161 – 10.745	3.221^a^	1.054 – 9.848
BMI(Kg/m.^2^)				
Underweight (< 19.0)	1		1	
Normal weight (19.0—< 25.0)	1.126	0.615 – 2.062	1.158	0.37 – 2.106
Overweight (25.0—< 30.0)	1.096	0.597 – 2.012	1.108	0.606 – 2.025
Obese (> = 30.0)	0.995	0.536 – 1.846	1.057	0.569 – 1.963
Baseline ART regimen				
AZT-3TC-NVP	1		1	
AZT-3TC-EFV	0.888	0.616 – 1.281	0.976	0.675 – 1.410
TDF-3TC-NVP	1.384	0.858 – 2.230	1.613	0.993 – 2.618
TDF-3TC-EFV	0.947	0.674 – 1.329	1.400	0.916 – 2.138
Other	0.599	0.335 – 1.072	1.075	0.555 – 2.082
No family history of HTN	1		1	
Positive family history of HTN	1.083	0.771 – 1.521	1.069	0.761 – 1.500
No history of stroke	1		1	
Positive History of stroke	0.836	0.401 – 1.743	0.763	0.368 – 1.579
No history of CKD	1		1	
Positive History of CKD	1.420	0.412 – 4.888	1.369	0.405 – 4.629
Diabetes Mellitus				
Non-diabetic	1		1	
Diabetic	1.103	0.774 – 1.573	1.098	0.766 – 1.574
Non-smoker	1		1	
Smoker	1.693	1.051 – 2.727	1.755	1.087 – 2.835

*CI* Confidence intervals, *CKD* Chronic kidney disease, *BMI*-Body mass index

^a^ significant odds ratio

## Discussion

In this study, we aimed to use the BCW framework to adapt a WHO HEARTS-based implementation strategy to integrate HTN management into HIV care through stakeholder engagement and local contextualization. Using this strategy, our HTN control among PLHIV and HTN increased from 9% at baseline to 74% at six months. The HEARTS technical packages for CVD control were introduced to improve HTN control in multiple settings [13–17]. However, to our knowledge, this is the first study to adapt the WHO HEARTS technical packages to integrate HTN management into a clinical setting for HIV care.

Among the HEARTS components, we believe that successful HTN/HIV integration in our setting was and will be hinged on the following: a simple, stepwise HTN protocol [13], consistent access to HTN medicines [15], task shifting of HTN screening and treatment to lower cadres of healthcare providers [17], strengthening HTN monitoring and evaluation and training and mentoring of healthcare providers [37, 38].

Our implementation strategy was effective in improving HTN control at this large urban HIV clinic. At baseline, the clinic had very nearly met or exceeded all three UNAIDS 90–90-90 goals regarding ART initiation, retention in care and viral suppression. In addition, the clinic had already achieved universal BP screening for all adult PLHIV, demonstrating a high prevalence of HTN (24.3%) [21, 22]. Despite a high prevalence of HTN, initiation of HTN treatment, retention, and HTN control were suboptimal (Fig. 4A). Similarly stark findings have been identified in other parts of SSA and in our previous studies in rural Uganda [21, 39]. Of the 3,892 hypertensive PLHIV at the clinic, we enrolled 1,015 patients into integrated HTN/HIV care. The remaining 2,877 patients declined enrollment. The major reason for non-enrollment was patients who were already taking other anti-hypertensive medications either from other health facilities or bought from retail pharmacies. At six months after implementing the integrated HTN/HIV care, our program had all the 1,015 enrolled hypertensive PLHIV receiving integrated HTN/HIV care. In line with the WHO and MoH HIV test and treat policy [25, 26], all enrolled PLHIV with HTN were initiated or maintained on ART and HTN medicines. Among the patients in our cohort, retention in HIV care, viral load monitoring and HIV control were all sustained above 95% both at baseline and six months (Fig. 4). These findings suggest that integrated HTN/HIV care sustains an optimal HIV care cascade.

The integrated HTN/HIV program initiated all hypertensive PLHIV in the cohort to HTN treatment, retained almost all of them in care, and routinely monitored most during each clinic visit. Importantly, HTN control at six months, a HEARTS Core Indicator, reached 74% [18]. Similarly high levels of HTN control were registered in the Kaiser Permanente HTN program of North California, which utilized a stepwise HTN protocol that had a thiazide diuretic as first line medication [40, 41]. This level of HTN control among PLHIV and the general population has not been achieved in SSA [21, 39]. In Uganda, a community-based epidemiologic study found a HTN control rate of 2.2% [42]. Our cohort participants were older and had been on ART longer than non-enrolled patients. These factors may have contributed to better adherence to both ART and HTN medications and hence better HTN control.

In our study, the rate of attrition from HTN care was low at 1.5%. In addition, there were few side effects of BP medications prescribed in our study, further supporting the safety and feasibility of our approach.

The medicines on our protocol, which we procured from a pharmaceutical access program, were available to the private not for profit sector and not yet adopted by the public health facilities. Further scale up of these low priced and effective agents will require including them into the essential medicines list of Uganda so that patients in the public health facilities can benefit. Our protocol has been adopted by the Uganda MoH and integrated into guidelines to integrate hypertension management into HIV care for national scale up.

Finally, patients who had been on ART for more than two years were more likely to achieve HTN control than those with shorter ART durations. Similar findings have been replicated in HIV programs across SSA regarding HIV and HTN control [43–47]. In this patient population, adherence to HIV medication is likely to predict adherence to antihypertensives. This was further emphasized by the better HTN control among patients who had achieved a suppressed HIV viral load which resulted from high levels of ART adherence. Additionally, the effect of a long duration of ART may be partly confounded by age [47–49].

### Limitations

Due to supply chain limitations, hydrochlorothiazide was not supplied until after the six-month follow up. Long-term follow up of our cohort will determine the added effectiveness of hydrochlorothiazide. We used a pre-post study design and described a six-month trend of hypertension control in our cohort after introducing the implementation strategy. This minimized the influence of autocorrelation in the follow up period. However, we did not analyze the pre-intervention trend because we did not collect that data. Therefore, baseline results may be affected by autocorrelation and seasonality. In this study, we did not conduct random sampling of cohort participants. The convenience sampling renders findings of this study less generalizable among diverse populations. Additionally, the pre-intervention assessment of BP could have sensitized patients to seek better care for HTN. This could confound the achievement and predictors of HTN control at six months. We were unable to control for unmeasured confounders since the study lacked a control group. Despite these limitations, the outcomes reported provide preliminary evidence for the effectiveness of an adapted HEARTS intervention on HTN control among PLHIV.

## Conclusions

An adapted WHO HEARTS-based implementation strategy at a large, urban HIV center facilitates integration of HTN and HIV care and improves HTN outcomes while sustaining HIV control. Further implementation research of the HEARTS strategy is needed to study HTN/HIV integration in varied clinical settings among diverse populations.

## Data Availability

The datasets used and/or analyzed during the current study are available from the corresponding author on reasonable request.

## Abbreviations

COM-B: Capability, Opportunity, Motivation and Behavior
HTN: Hypertension
PLHIV: Persons Living with HIV
CVD: Cardiovascular Disease
NCD: Non-Communicable Disease
ART: Antiretroviral Therapy
WHO: World Health Organization
MoH: Ministry of Health
